# Atherosclerosis of the right posterior hepatic artery in a patient with hilar cholangiocarcinoma undergoing left trisectionectomy: a case report of a therapeutic pitfall

**DOI:** 10.1186/s12893-018-0415-2

**Published:** 2018-09-24

**Authors:** Yuichi Goto, Satoki Kojima, Yoriko Nomura, Daisuke Muroya, Syoichiro Arai, Hisamune Sakai, Ryuichi Kawahara, Toru Hisaka, Yoshito Akagi, Hiroyuki Tanaka, Koji Okuda

**Affiliations:** Division of Hepatobiliary and Pancreatic Surgery, Department of Surgery, Kurume University School of Medicine, 67 Asahi-machi, Kurume, 8300011 Japan

**Keywords:** Hepatic artery, Stenosis, Atherosclerosis, Hilar cholangiocarcinoma, Pyogenic liver abscess

## Abstract

**Background:**

We experienced a rare case of benign arterial stricture of the right posterior hepatic artery (RPHA) caused by atherosclerosis in a patient with hilar cholangiocarcinoma.

**Case presentation:**

A 75-year-old man was referred to our hospital for the detailed investigation of serum hepatobiliary enzyme elevation. The patient had a history of hypertension, type 2 diabetes mellitus, and an operative history of coronary artery bypass grafting 10 years before. Endoscopic retrograde cholangiography found strictures of the right and left hepatic ducts with involvement of right anterior and posterior bile ducts. Adenocarcinoma was evident by brush cytology. We diagnosed these findings as hilar cholangiocarcinoma and planned left trisectionectomy including bile duct reconstruction. Although the tumor and RPHA were not adjacent, preoperative multidetector computed tomography revealed a stricture of the RPHA that was 5.6 mm in length. We suspected that atherosclerosis caused the stricture, and we performed digital subtraction angiography and intravascular ultrasonography that showed stricture of the RPHA accompanied by thick plaques in the arterial wall. We placed a bare-metal stent in the RPHA and then performed left trisectionectomy. Since this patient developed bile leakage postoperatively, percutaneous drainage was performed. The bile leakage was successfully controlled, and the patient was discharged 3 months after surgery. Unfortunately, 4 months after hepatectomy, he was re-hospitalized with multiple pyogenic liver abscesses. We performed intensive multimodal treatment for the liver abscesses and stabilized the disease; however, we eventually lost this patient due to liver failure 14 months after surgery.

**Conclusion:**

To the best of our knowledge, there is no previous literature on atherosclerosis of the RPHA, which was evident preoperatively in our case. Because arterial complications may lead to critical biliary complications in patients who undergo left trisectionectomy, we first performed prophylactic arterial stent placement. We speculate that existing chronic microscopic injury of the peribiliary plexus might have caused the liver abscesses. We successfully diagnosed atherosclerosis of the RPHA preoperatively. However, further investigation of patients is warranted to determine if left trisectionectomy is contraindicated in these patients.

## Background

With the increasing number of elderly surgical candidates, underlying cardiovascular complications are an important factor for assessing the risk of surgery [1]. There is no previous literature on benign stricture of the right posterior hepatic artery (RPHA) caused by atherosclerosis. We report an extremely rare case of hilar cholangiocarcinoma, in which there was atherosclerosis of the RPHA. This patient was managed preoperatively by bare-metal stent placement followed by left trisectionectomy including Roux-en-Y choledochojejunostomy.

## Case presentation

A 75-year-old male patient was referred to our hospital for the detailed investigation of elevated serum hepatobiliary enzymes. The patient had a history of hypertension, type 2 diabetes mellitus (DM) with diabetic nephropathy, malignant otitis externa, and an operative history of emergent coronary artery bypass grafting (CABG) 10 years before for myocardial infarction. During cardiac surgery, the right gastric artery, which was bifurcated from the common hepatic artery, was anastomosed to the posterior descending artery by the ante-gastric route. The height and weight of the patient was 1.62 m and 65 kg, respectively. The performance status of the patient assessed by the Eastern Cooperative Oncology Group was Grade 1. The results of preoperative laboratory testing were as follows: white blood cell count, 5,900 cells/μL; red blood cell count, 478 × 10^4^ cells/μL; serum hemoglobin concentration, 10.3 g/dL; serum platelet count, 16.6 × 10^4^ platelets/μL; serum aspartate aminotransferase, 40 IU/L; serum alanine aminotransferase, 32 IU/L; serum alkaline phosphatase, 639 IU/L; serum gamma glutamic transpeptidase, 297 IU/L; total serum bilirubin, 0.66 mg/dL; serum albumin, 3.91 g/dL; serum C-reactive protein, 0.05 mg/dL; prothrombin time (%), 114%; hemoglobin A1c, 7.6%; indocyanin green retention rate after 15 min, 4.0%. The levels of serum carcinoembrionic antigen and carbohydrate antigen 19–9 were elevated at 2.5 ng/mL and 35.8 U/mL, respectively.

**Fig. 1:**
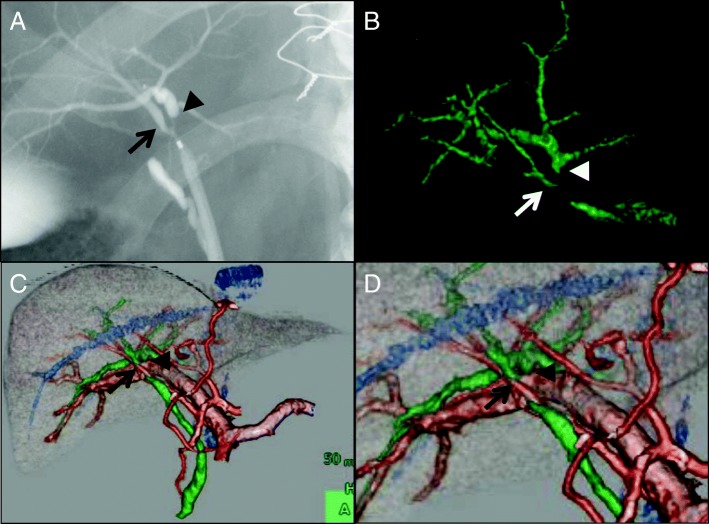
Endoscopic retrograde cholangiography (**a**) and 3-dimensional CT cholangiography (**b**, **c**, **d**) reveals stenosis of the common hepatic duct that was 20 mm in length including the right and left hepatic ducts, with involvement of the right anterior (arrow) and posterior (arrow head) bile duct bifurcation. The pattern of confluence of the right posterior hepatic artery is of the supra-portal type. The left hepatic duct is completely occluded by cancer and could not be identified

Endoscopic retrograde cholangiography and 3-dimensional computed tomography cholangiography found abrupt narrowing of the common hepatic duct that was 20 mm in length including the right and left hepatic ducts, with involvement of the right anterior and posterior bile duct bifurcations (Fig. 1a-d). Adenocarcinoma was evident by the cytological findings, and this was confirmed by brush cytology. Contrast enhanced computed tomography (CECT) showed no distant metastases or lymph node metastases. We diagnosed these findings as Bismuth type 4, T2N0M0 Stage II hilar cholangiocarcinoma. According to the preoperative imaging findings, the left bile duct was completely occluded by cancer, there was narrowing of right hepatic duct and anterior sector bile duct, and only the right posterior bile duct was free from the margin. Therefore, we planned left trisectionectomy, extrahepatic bile duct resection, and Roux-en-Y choledochojejunostomy.

**Fig. 2:**
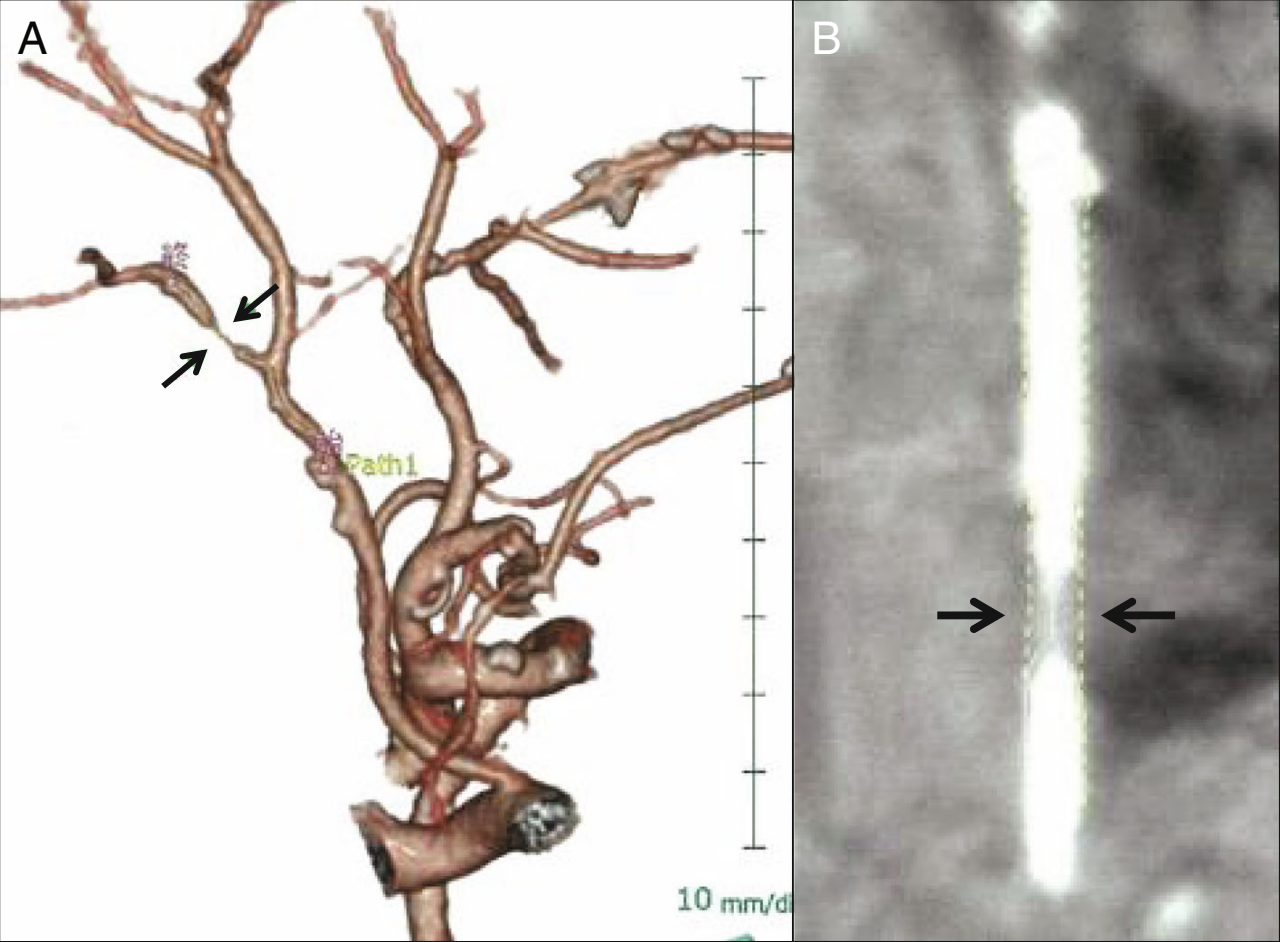
Three-dimensional multidetector computed tomography (MDCT) (**a**) and curved planner reconstruction of MDCT (**b**) showed stricture of the right posterior hepatic artery that was 5.6 mm in length (arrows)

**Fig. 3:**
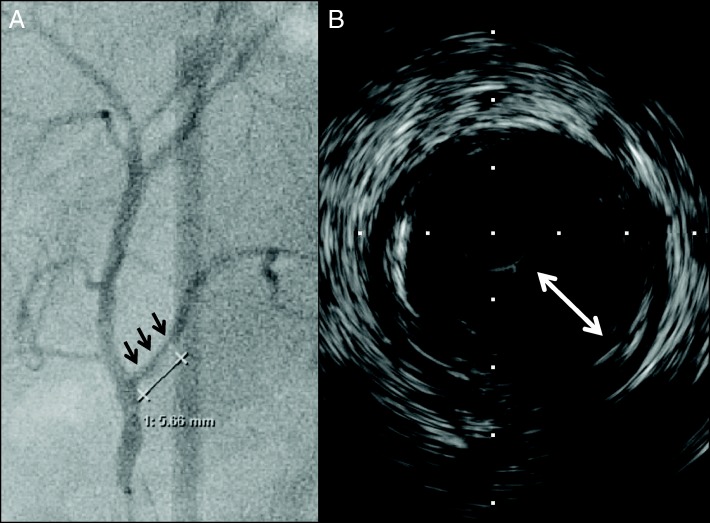
Digital subtraction angiography (**a**) shows stricture of the right posterior hepatic artery (arrows), and thick plaques (double-head arrow) in the arterial intima were observed by intravascular ultrasonography (**b**)

**Fig. 4:**
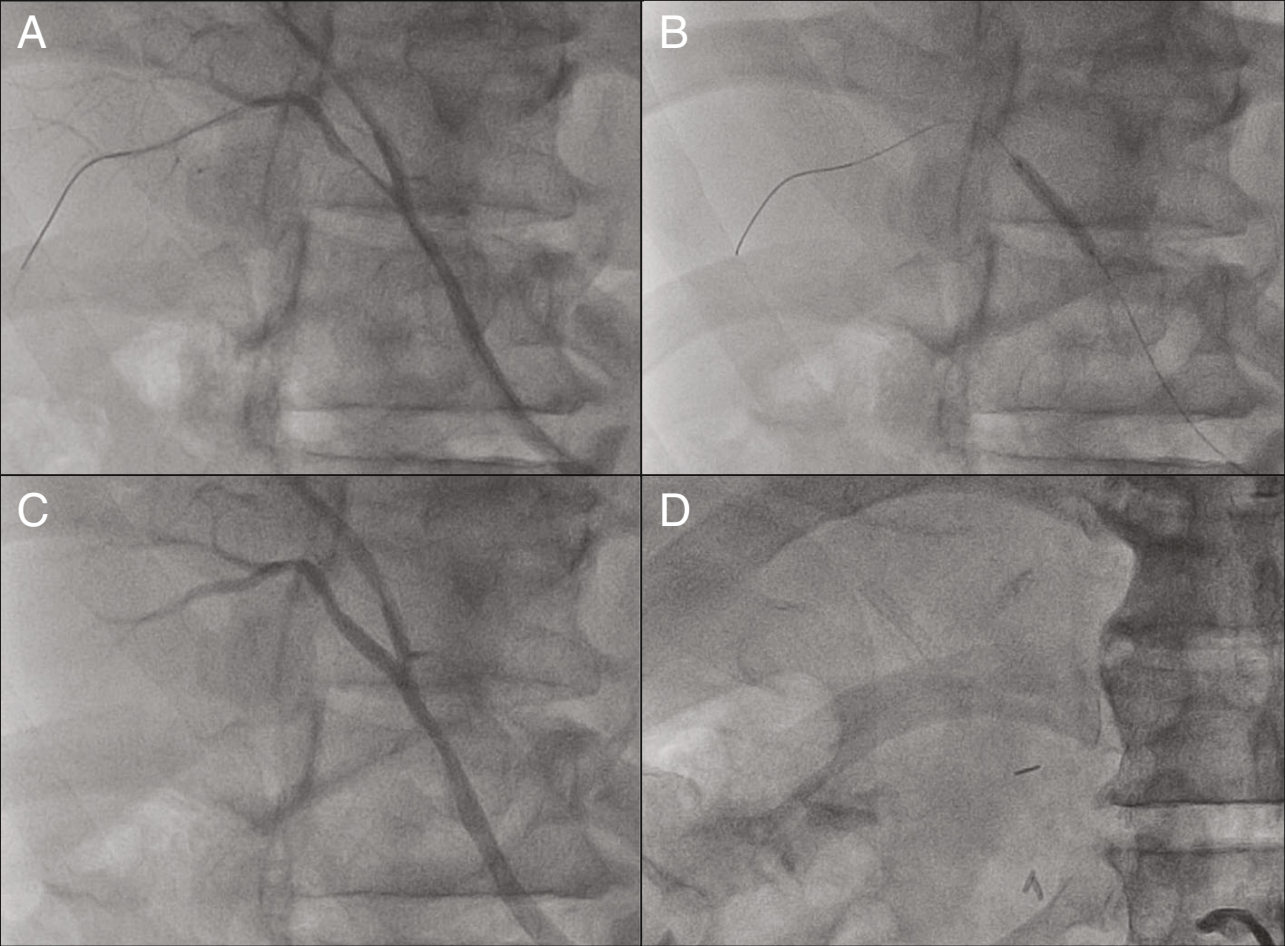
Digital subtraction angiography before (**a**) and after the percutaneous balloon arterioplasty (**b**). The stricture of the right posterior hepatic artery was improved after placement of the bare-metal stent (**c**, **d**)

CECT also showed that the right hepatic artery was bifurcated from the supra mesenteric artery, and the left hepatic artery was dominant from the celiac artery. The bile duct, which was involved in the cholangiocarcinoma, and the RPHA were not adjacent (the distance between them was 8 mm) because the pattern of confluence of the right posterior hepatic duct was of the supra-portal type, although the narrowing of the RPHA was 5.6 mm in length. Because this patient had multiple cardiovascular risk factors including a history of CABG, we suspected that the narrowing of the RPHA was a benign stricture caused by atherosclerosis. Arterial imaging with 3-dimensional multidetector computed tomography (MDCT) (Fig. 2a) and curved planner reconstruction (CPR) of MDCT also showed stricture of the RPHA (Fig. 2b). We performed digital subtraction angiography (DSA) and intravascular ultrasonography (IVUS). DSA showed stricture of the RPHA (Fig. 3a), and thick plaques in the arterial intima were evident on IVUS (Fig. 3b). According to these findings, we diagnosed stricture of the RPHA due to atherosclerosis, and we performed percutaneous balloon arterioplasty followed by bare-metal stent placement to prevent postoperative biliary complications (Fig. 4a-d). His clinical course after stent placement was uneventful, and we performed left trisectionectomy, extrahepatic bile duct resection, and Roux-en-Y choledochojejunostomy 51 days after stent placement.

**Fig. 5:**
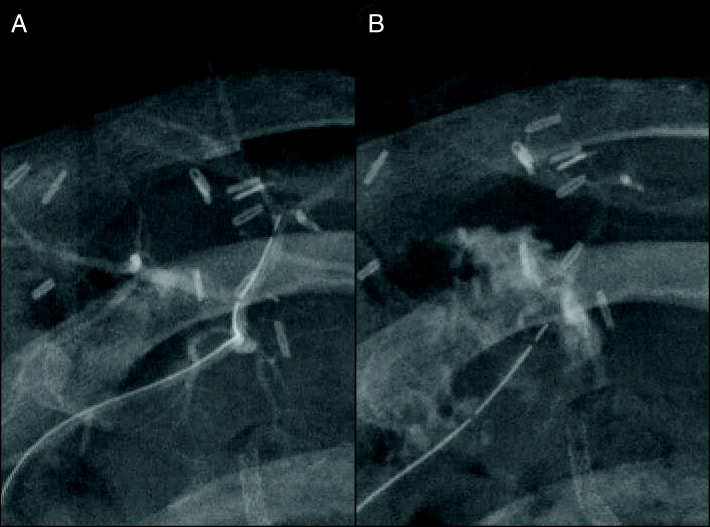
Cholangiography of the bile duct drainage tube revealed no stenosis of the choledochojejunostomy. There was no dilatation of the posterior bile duct (**a**), and the passage of contrast media was smooth without retention after removal of the drainage tube (**b**)

**Fig. 6:**
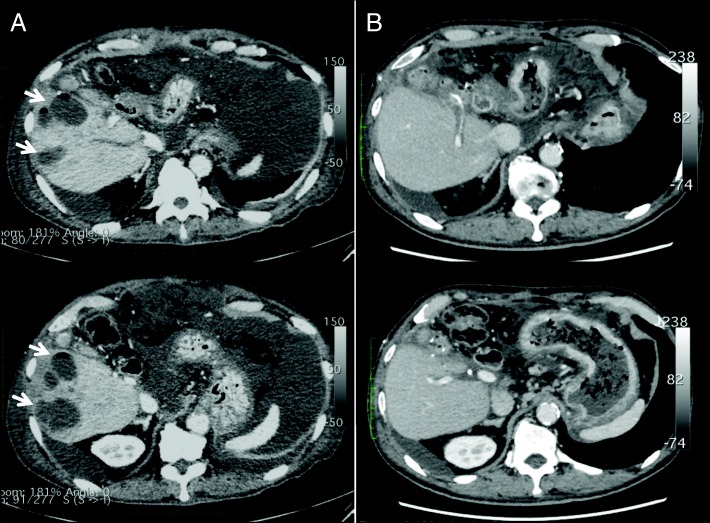
Multiple pyrogenic liver abscesses (arrows) in the remnant liver (**a**). Liver abscesses disappeared after intensive treatment (**b**)

Since this patient developed bile leakage postoperatively, percutaneous drainage was performed. The bile leakage was successfully controlled without stenosis of the choledochojejunostomy (Fig. 5a, b), and the patient was discharged 3 months after surgery. Unfortunately, 4 months after hepatectomy, he was re-hospitalized with multiple pyogenic liver abscesses (Fig. 6a). *Klebsiella pneumoniae* was identified from the liver abscess, and we performed intensive multimodal treatment for the pyogenic liver abscesses. Although we stabilized the disease (Fig. 6b), we eventually lost this patient due to liver failure 14 months after surgery.

## Discussion and conclusion

Left trisectionectomy is the treatment of choice for patients with Bismuth type 4 hilar cholangiocarcinoma. Although the safety of this procedure has been increasing, the reported incidence of postoperative complications is still high [2–4]. Because the RPHA is the only remaining artery that supplies blood flow to the intrahepatic bile duct after left trisectionectomy, preoperative assessment of RPHA is important. To the best of our knowledge, there is no previous literature on atherosclerosis of the RPHA, and there is no consensus about how it should be managed.

Since our patient had hilar cholangiocarcinoma that was accompanied by benign stricture of the RPHA due to atherosclerosis, we performed stent placement before left trisectionectomy. To assess vascular anatomy and liver volume, we performed 128-slice MDCT. MDCT in combination with CPR analysis was quite sensitive to identify stenosis of the RPHA, and atherosclerosis was confirmed by IVUS. Because it has been reported that atherosclerosis of the hepatic artery is related to arterial dissection, aneurysm, and thrombosis [5], we performed prophylactic stent placement to prevent postoperative arterial complications. Although the diagnostic and therapeutic procedures were successful, the patient suffered from multiple pyogenic liver abscesses 4 months after liver surgery. The incidence of postoperative pyogenic liver abscess in patients with hilar cholangiocarcinoma undergoing major hepatectomy is reported to range from 20 to 37.5% [4, 6]. Concomitant resection of the hepatic artery and/or portal vein accompanied by vascular reconstruction is associated with a high risk of liver abscess after surgery [6]. Biliary disorders including cholecystolithiasis, choledocholothiasis, and hepatolithiasis are also related to liver abscess [6], although they were not evident in our case.

From an anatomical perspective, the intrahepatic biliary ducts are surrounded by a rich microvascular network, which is called the peribiliary plexus [7]. Some experimental studies suggested that there is an arterioportal communication through the peribiliary plexus [8]. The peribiliary plexus represents a collateral source of arterial blood to the liver when the hepatic artery is occluded [9]. We also performed Doppler ultrasonography to measure the resistive index of the RPHA and confirmed favorable arterial blood flow before and after stent placement including liver surgery. When the patient was re-hospitalized for pyogenic liver abscesses, there was no sign of arterial complications including stent occlusion. Since arterial flow through the RPHA was preserved, we speculate that microscopic injury of the peribiliary plexus including ischemic changes possibly due to atherosclerosis might have caused his pyrogenic liver abscesses. Since DM has also been reported to be associated with liver abscess in patients with hilar cholangiocarcinoma who undergo surgery, it possible that DM could have influenced the outcome of our patient, even though his DM was controlled by insulin therapy [10, 11]. In addition, our patient also suffered from refractory peptic ulcer and was treated with a proton-pump inhibitor (PPI). Prolonged use of a PPI has been reported to increase the risk and mortality of pyogenic liver abscess [12]. Since our patient had vascular complications as well as other comorbidities that are associated with liver abscess, it possible that all of these factors could have affected the outcome.

As for the stent placement, a drug-eluting stent might be better than a bare-metal stent for reducing the risk of stent thrombosis [13]. One reason that we selected a bare-metal stent was that we had to cover the artery from the right hepatic artery to the RPHA without disturbing arterial flow of the right anterior hepatic artery, since the stenosis of the RPHA extended to the bifurcation of the anterior and posterior hepatic arteries. Another reason was the timing of liver surgery. After stent placement, double antiplatelet therapy is routinely administered, and the American College of Cardiology and American Heart Association guidelines recommended postponing elective non-cardiac surgery until 30 days after bare-metal stent placement and 6 months after drug-eluting stent placement [14]. We finally performed left trisectionectomy 51 days after placement of the bare-metal stent, and we do not think it would have been possible to wait for 6 months, since delay of surgery in patients with hilar cholangiocarcinoma will reduce the cure rate due to disease progression.

There is a limitation of our report. Since there was no autopsy in this patient after his death, histopathological assessment of the remnant liver was not performed to determine if there was microvascular injury of the peribiliary plexus due to atherosclerosis.

We successfully diagnosed atherosclerosis of the RPHA preoperatively by multiple imaging modalities and performed stent placement to prevent postoperative complications; however, we lost the patient due to the pyogenic liver abscesses. Further investigations are warranted to confirm if atherosclerosis of the RPHA is a potential risk factor for liver abscess and to determine if left trisectionectomy for hilar cholangiocarcinoma is contraindicated in these patients.

## Abbreviations

CABG: Coronary artery bypass graft
CECT: Contrast enhanced computed tomography
DM: Diabetes mellitus
DSA: Digital subtraction angiography
ERCP: Endoscopic retrograde cholangiography
IVUS: Intravascular ultrasonography
MDCT: Multidetector computed tomography
RPHA: Right posterior hepatic artery
